# Serum glycated albumin is associated with in-stent restenosis in patients with acute coronary syndrome after percutaneous coronary intervention with drug-eluting stents: An observational study

**DOI:** 10.3389/fcvm.2022.943185

**Published:** 2022-09-27

**Authors:** Xiao Long Lin, Qiu Yu Li, Dong Hui Zhao, Jing Hua Liu, Qian Fan

**Affiliations:** Center for Coronary Artery Disease, Beijing Anzhen Hospital, Capital Medical University, Beijing Institute of Heart, Lung, and Blood Vessel Diseases, Beijing, China

**Keywords:** glycated albumin (GA), in-stent restenosis (ISR), drug-eluting stents (DESs), acute coronary syndrome (ACS), percutaneous coronary intervention (PCI)

## Abstract

**Background:**

Previous studies have confirmed the predicted value of serum glycated albumin (GA) in atherosclerotic cardiovascular disease. However, the relationship between GA and the development of in-stent restenosis (ISR) after drug-eluting stent (DES) implantation has not been verified in patients with acute coronary syndrome (ACS).

**Materials and methods:**

In this study, 797 patients diagnosed with ACS who underwent re-coronary angiography more than 6 months after the first successful DES-based percutaneous coronary intervention (PCI) were eventually included. Patients were categorized into two groups based on the median GA levels of 14.94%. Moreover, multivariate logistic regression analysis models and the net reclassification improvement and integrated differentiation improvement risk models were constructed to assess the relationship between the GA and DES-ISR in patients with ACS.

**Results:**

The GA was significantly associated with an increased risk of DES-ISR, upon adjusting for confounding factors (as nominal variate: OR 1.868, 95% CI 1.191–2.932, *P* = 0.007; as continuous variate: OR 1.109, 95% CI 1.040–1.183, *P* = 0.002). The addition of GA to a baseline risk model had an incremental effect on the predictive value for DES-ISR (AUC: GA vs. baseline model, 0.714 vs. 0.692, comparison *P* = 0.017; category-free net reclassification improvement (NRI) 0.080, *P* = 0.035; integrated discrimination improvement (IDI) 0.023, *P* < 0.001).

**Conclusion:**

GA level was significantly associated with a high risk of DES-ISR in patients with ACS treated with PCI. Moreover, the addition of the GA to a baseline risk model has an incremental effect on the predictive potential for DES-ISR.

## Introduction

Acute coronary syndrome (ACS) is one of the most common diseases threatening human health and life span worldwide (1). Due to percutaneous coronary intervention (PCI) reducing the invasiveness and shortening the operation time, it has become the primary treatment strategy for coronary heart disease (CAD) (2). Despite significant advances in interventional techniques, drug therapy, and the use of drug-eluting stents (DES), the incidence of in-stent restenosis (ISR) has declined. However, the incidence of DES-ISR remains high, and it is reported that the occurrence rate of ISR ranges from 3 to 20% after coronary DES implantation (3). Patients with DES-ISR are more likely to have symptoms of unstable angina than patients with *de novo* stenosis (4) and have a higher prevalence of acute myocardial infarction (5). Given the high incidence of DES-ISR and the adverse events associated with ISR, the search for biomarkers to predict DES-ISR is still of great significance and practical clinical value.

Diabetes mellitus (DM) has been identified as an independent risk factor for DES-ISR (6), and glycated albumin (GA) is closely related to the prevalence of DM (7). In a report published in 1979 (8), Dolhofere and Weiland first found elevated GA in patients with diabetes. After 8 years, other researchers reported that GA was associated with glycated hemoglobin (HbA1c) in patients with diabetes and was suggested as another clinical indicator for blood glucose monitoring (9, 10). In the comparative study of GA and HbA1c, the researchers found that GA reflects blood glucose control for the previous 2–3 weeks, while HbA1c demonstrates glycemic control status in the preceding 2–3 months (11, 12). Furthermore, GA is superior to HbA1c for monitoring blood glucose in some clinical situations (13), and multiple studies have confirmed the predicted value of GA in ASCVD (14). However, the relationship between the GA and the ISR has been rarely reported. Only the study by Lu et al. (15) showed that increased GA was associated with ISR in Chinese patients with diabetes. However, this study only evaluated ISR occurrence in patients with diabetes who received bare-metal stent implantation, and the sample size is small. Additionally, the interventional techniques and medical therapies were more backward, and ISR incidence was much higher than now. Due to these limitations, the results of this study do not reflect the proper relationship between GA and ISR.

At present, this is the era of DES implantation during PCI. But, to date, the relationship between GA and the development of DES-ISR has not been verified in patients with ACS. Moreover, studies comparing various glycemic indexes for predicting the occurrence of DES-ISR are lacking. Therefore, we intend to investigate GA for its predictive value for ISR in patients with ACS after DES-based PCI.

## Materials and methods

### Study population

This is a single-center, retrospective, observational cohort study. From January 2019 to June 2021, 797 consecutive patients diagnosed with ACS undergoing PCI at Beijing Anzhen Hospital, Capital Medical University, were enrolled. The main exclusion criteria were as follows: (1) age less than 18 years, (2) missing clinical or coronary angiography data, (3) PCI failure, PCI-related complications or only PTCA, (4) underwent follow-up angiography after successful PCI less than 6 months, and (5) chronic inflammatory disease, malignant tumor or severe hepatic dysfunction (Supplementary Figure 1). This study was performed according to the Helsinki Declaration of Human Rights (2000) and approved by the Clinical Research Ethics Committee of Beijing Anzhen Hospital, Capital Medical University. Alternatively, written informed consent was obtained from all patients.

### Angiographic analysis and stent implantation

Coronary angiography was performed using the standard Judkins technique through the radial or femoral approach. Coronary intervention and stent implantation were performed according to current practice guidelines (16). The stent material was G2-DESs, which included zotarolimus-eluting stents, domestic sirolimus-eluting stents, and everolimus-eluting stents. Coronary angiographic analysis and stent selections were performed by experienced interventional cardiologists. Before the procedure, all patients received aspirin (300 mg loading dose followed by 100 mg/day) combined with clopidogrel (300 mg loading dose followed by 75 mg/day) or ticagrelor (180 mg loading dose followed by 90 mg 2 times/day). During the procedure, patients received anticoagulation with heparin (100 IU/kg) to maintain an activated clotting time >250 s.

### Demographic and clinical data

Patients’ data of demographic and clinical characteristics regarding age, sex, BMI, systolic blood pressure (SBP), diastolic blood pressure (DBP), smoking status, medical history, and left ventricular ejection fraction (LVEF) were collected from Beijing Anzhen Hospital’s medical information recording system. Meanwhile, we also recorded laboratory examinations, including the white blood cell count, hemoglobin, platelet count, high sensitivity-C reactive protein (hs-CRP), eGFR, uric acid, FBG, HbA1c, GA, total cholesterol (TC), low-density lipoprotein-C (LDL-C), high-density lipoprotein cholesterol (HDL-C), and triglyceride (TG), which were determined at the central laboratory of Beijing Anzhen Hospital. The GA levels were determined by the enzymatic method using the Lucica GA-L kit (Asahi Kasei Pharma, Tokyo) (17). The value of GA is expressed as a percentage of the total albumin concentration. Furthermore, two experienced investigators recorded coronary angiogram data such as stent diameter, stent length, and stenosis percent at baseline and follow-up for coronary angiography analysis.

### Disease definitions

In-stent restenosis was defined as ≥50% lumen restenosis of the artery within 5 mm proximal or distal of the stent segment or stent region after PCI, which was determined by angiography (18). The target lesion was the most severe narrowing vessel identified by angiographic appearance with electrocardiographic (ECG) changes. Multivessel disease (MVD) was defined as diameter stenosis of ≥50% occurring in 2 or more vessels.

### Statistical analysis

Continuous variables were presented as the mean ± standard deviation is consistent with a normal distribution, otherwise as to the median and interquartile range (IQR). Categorical variables were expressed as numbers and percentages. A one-way ANOVA or Mann-Whitney U-test was used to analyze differences in continuous variables. The Pearson chi-square test (Pearson X^2^ test), Fisher’s exact test or the Cochran-Armitage trend test was used to analyze categorical variables. The admission values of serum GA were divided into two groups based on median GA to stratify the incidence rates of DES-ISR. Univariate and multivariate logistic regression analyses were used to estimate the incidence of ISR. The baseline variables that showed *p* < 0.05 in univariate analysis were included in the multivariate analysis. In multivariate logistic regression analysis, three models were established for evaluating the prognosis of GA in DES-ISR: Model 1, adjusted for age, BMI, and diabetes; Model 2, adjusted for variates in Model 1 and previous MI, previous PCI, FBG, HbA1c, LDL-C, and LVEF; and Model 3, adjusted for variates in Model 2 and one-vessel disease, multiverse/LM disease, number of stents, multiple stents (≥2), the total length of stents, and minimal stent diameter. The analysis results were presented as odds ratios (ORs) and 95% confidence intervals (CIs). To verify the robustness of our results, subgroup analyses were performed to explore the association between GA and DES-ISR.

Receiver operating characteristic (ROC) curves were constructed, and the area under the curve (AUC) was calculated to reflect the GA’s predictive value for the developing DES-ISR. Meanwhile, to evaluate whether introducing the GA into the baseline risk model could improve the predictive value, the C-statistic was compared using Delong’s test (19). The net reclassification improvement (NRI) and integrated differentiation improvement (IDI) risk models were used to further evaluate the incremental predictive value of GA. Data were analyzed using the IBM SPSS statistics 24 and R software. For all comparisons, two-sided probability values <0.05 indicated statistical significance.

## Results

### Baseline characteristics

A total of 797 patients diagnosed with ACS who underwent re-coronary angiography for more than 6 months after the first successful DES-based PCI were eventually included in this study. As shown in Table 1, the male-to-female ratio was approximately 3:1, and the mean age was 59.03 ± 9.55 years. Among these populations, 394 (49.4%) participants were previous or current smoking, 525 (65.9%) participants were hypertension, 288 (36.1%) participants were diabetes, 561 (70.4%) participants were dyslipidemia, and 211 (26.5%) participants were the previous PCI. Regarding coronary angiography and PCI, 84.1% of the lesions were multiverse/left main (LM) diseases. The left anterior descending artery (LAD) and right coronary artery (RCA) accounted for nearly 79% of target vessel interventions, 36.4% of patients had two or more stents implanted, and half of the stent materials were DES sirolimus.

**TABLE 1:** Baseline characteristics of the study patients.

	Total population (*n* = 797)	Lower GA(≤14.94; *n* = 399)	Higher GA(>14.94; *n* = 398)	*P*-value
Age, years	59.03 ± 9.55	56.68 ± 9.59	61.38 ± 8.92	<0.001
Male, n (%)	600 (75.3)	320 (80.2)	280 (70.4)	0.002
BMI, kg/m^2^	26.49 ± 3.27	26.77 ± 3.49	26.21 ± 3.01	0.017
Systolic BP, mmHg	128.91 ± 16.64	127.58 ± 16.23	130.25 ± 16.96	0.024
Diastolic BP, mmHg	76.82 ± 10.92	77.84 ± 11.09	75.80 ± 10.67	0.008
Heart rate, bpm	70.97 ± 9.45	70.57 ± 9.73	71.37 ± 9.16	0.234
**Medical history, n (%)**				
Previous or current Smoking, n (%)	394 (49.4)	209 (52.4)	185 (46.5)	0.111
Previous or current Drinking, n (%)	251 (31.5)	134 (33.6)	117 (29.4)	0.232
Hypertension, n (%)	525 (65.9)	258 (64.7)	267 (67.1)	0.518
Diabetes, n (%)	288 (36.1)	33 (8.3)	255 (64.1)	<0.001
Dyslipidemia, n (%)	561 (70.4)	265 (66.4)	296 (74.4)	0.017
Previous MI, n (%)	132 (16.6)	61 (15.3)	71 (17.8)	0.382
Previous PCI, n (%)	211 (26.5)	88 (22.1)	123 (30.9)	0.006
Previous Stroke, n (%)	84 (10.5)	38 (9.5)	46 (11.6)	0.412
Laboratory values at hospital admission				
WBC count, ×10^9^/L	7.54 ± 2.48	7.64 ± 2.90	7.44 ± 1.97	0.267
Hemoglobin, g/L	141.76 ± 15.13	144.19 ± 14.50	139.33 ± 15.38	<0.001
Platelet count, ×10^9^/L	227.18 ± 60.14	231.50 ± 56.18	222.84 ± 63.64	0.042
Hs-CRP, mg/L	1.54 (0.64,3.96)	1.39 (0.60,3.54)	1.71 (0.70,4.43)	0.035
eGFR, mL/min	92.97 ± 16.55	95.08 ± 16.66	90.85 ± 16.19	<0.001
Uric acid, umol/L	348.96 ± 98.49	365.04 ± 96.54	332.83 ± 97.90	<0.001
FBG, mmol/L	6.72 ± 2.47	5.68 ± 1.19	7.75 ± 2.95	<0.001
HbA1c, %	6.61 ± 1.34	5.88 ± 0.75	7.34 ± 1.40	<0.001
TC (mmol/L)	3.97 ± 0.91	4.00 ± 0.93	3.94 ± 0.88	0.365
TG (mmol/L)	1.74 ± 1.22	1.73 ± 1.09	1.75 ± 1.34	0.825
LDL-C (mmol/L)	2.39 ± 0.81	2.43 ± 0.81	2.35 ± 0.80	0.170
HDL-C (mmol/L)	1.05 ± 0.25	1.05 ± 0.26	1.05 ± 0.24	0.889
LVEF (%)	61.57 ± 7.74	61.46 ± 7.91	61.68 ± 7.57	0.682
**Angiography**				
One-vessel disease, n (%)	132 (16.6)	64 (16.0)	68 (17.1)	0.763
Multivessel/LM disease, n (%)	670 (84.1)	339 (85.0)	331 (83.2)	0.551
Chronic total occlusion, n (%)	46 (5.8)	25 (6.3)	21 (5.3)	0.655
**Intervention**				
Target vessel, n (%)				
LM	21 (2.6)	15 (3.8)	6 (1.5)	0.078
LAD	334 (41.9)	162 (40.6)	172 (43.2)	0.499
LCX	146 (18.3)	66 (16.5)	80 (20.1)	0.227
RCA	291 (36.5)	155 (38.8)	136 (34.2)	0.194
Multiple stents (≥2)	290 (36.4)	146 (36.6)	144 (36.2)	0.963
Total length of stents, mm/patients	37.35 ± 23.31	37.65 ± 23.93	37.04 ± 22.70	0.711
Minimal stent diameter, mm	2.95 ± 1.27	3.04 ± 1.68	2.86 ± 0.64	0.046
DES-sirolimus, n (%)	424 (53.2)	210 (52.6)	214 (53.8)	0.802
DES-zotarolimus, n (%)	162 (20.3)	81 (20.3)	81 (20.4)	0.986
DES-everolimus, n (%)	208 (26.1)	105 (26.3)	103 (25.9)	0.952
**Clinical diagnosis**				
STEMI, n (%)	68 (8.5)	36 (9.0)	32 (8.0)	
NSTEMI, n (%)	69 (8.7)	38 (9.5)	31 (7.8)	
UA, n (%)	644 (80.8)	318 (79.7)	326 (81.9)	
**Medications in hospital, n (%)**				
Aspirin	797 (100.0)	399 (100.0)	398 (100.0)	>0.99
Clopidogrel/Ticagrelor	797 (100.0)	399 (100.0)	398 (100.0)	>0.99
Statin	797 (100.0)	399 (100.0)	398 (100.0)	>0.99
β-block	507 (63.6)	252 (63.2)	255 (64.1)	0.846
ACEI/ARB	351 (44.0)	175 (43.9)	176 (44.2)	0.975
Insulin	71 (8.9)	3 (0.8)	68 (17.1)	<0.001

BMI, body mass index; MI, myocardial infarction; PCI, percutaneous coronary intervention; WBC, white blood cell; Hs-CRP, high sensitivity c-reactive protein; eGFR, estimated glomerular filtration rate; FBG, fasting blood glucose; HbA1c, glycosylated hemoglobin A1c; TC, total cholesterol; TG, triglycerides; LDL-C, low-density lipoprotein cholesterol; HDL-C, high-density lipoprotein cholesterol; LVEF, left ventricular ejection fraction; LM, left main; LAD, left anterior descending artery; LCX, left circumflex artery; RCA, right coronary artery; DES, drug-eluting stents; STEMI, ST-segment elevation myocardial infarction; NSTEMI, non-ST-segment elevation myocardial infarction; UA, unstable angina; ACEI, angiotensin converting enzyme inhibitor; ARB, angiotensin receptor blocker; IQR, interquartile range. Values are presented as the mean ± SD, median (IQR) or number (%).

Based on the median GA, patients were divided into two groups (Table 1). As shown in Table 1, patients with a higher GA group showed higher age and systolic BP, diastolic BP, and lower body mass index (BMI), and had a higher proportion of women, with diabetes, dyslipidemia, and previous PCI. For laboratory values at hospital admission, lower levels of hemoglobin, estimated glomerular filtration rate (eGFR), uric acid, and higher levels of fasting blood glucose (FBG), and HbA1c were observed in the higher GA groups. In terms of CAG and PCI, patients in the higher GA group had a more complex lesion and more severe ISR (as shown in Supplementary Table 1).

At the same time, differences between the ISR group and the non-ISR group were analyzed (Table 2). As demonstrated in Table 2, age, BMI, DM, previous MI, and previous PCI were significantly higher in the ISR group than in the non-ISR group. Correspondingly, left ventricular ejection fraction (LVEF) was lower, whereas serum FBG, HbA1c, and GA were higher in subjects in the ISR group. The same results were observed in the follow-up lab measures. As shown in Supplementary Table 2, the follow-up lab measures, particularly LDL, FPG, HbA1C, and GA, decreased compared to the first pre-PCI period, which was associated with pharmacological treatment, but we still observed higher levels of these indicators in the ISR group than in the non-ISR group. Moreover, patients in the ISR group were more likely to suffer from multiple vessel disease, have two or more stents implanted, and have a longer total length of stents. As shown in Supplementary Table 3, compared with non-ISR, coronary artery lesions in the ISR group were more complex (such as the proportion with type C lesions is 64.9%), with longer lesion length and smaller reference diameter.

**TABLE 2:** Baseline characteristics of patients with and without ISR.

	Total population (*n* = 797)	Non-ISR group (*n* = 595)	ISR group (*n* = 202)	*P*-value
Age, years	59.03 ± 9.55	58.63 ± 9.80	60.19 ± 8.68	0.045
Male, n (%)	600 (75.3)	447 (75.1)	153 (75.7)	0.935
BMI, kg/m^2^	26.49 ± 3.27	26.35 ± 3.36	26.90 ± 2.95	0.038
Systolic BP, mmHg	128.91 ± 16.64	129.02 ± 16.43	128.60 ± 17.30	0.761
Diastolic BP, mmHg	76.82 ± 10.92	77.17 ± 11.08	75.79 ± 10.38	0.119
Heart rate, bpm	70.97 ± 9.45	70.95 ± 9.65	71.04 ± 8.86	0.904
**Medical history, n (%)**				
Previous or current Smoking, n (%)	394 (49.4)	291 (48.9)	103 (51.0)	0.667
Previous or current Drinking, n (%)	251 (31.5)	187 (31.4)	64 (31.7)	0.946
Hypertension, n (%)	525 (65.9)	398 (66.9)	127 (62.9)	0.339
Diabetes, n (%)	288 (36.1)	192 (32.3)	96 (47.5)	<0.001
Dyslipidemia, n (%)	561 (70.4)	417 (70.1)	144 (71.3)	0.815
Previous MI, n (%)	132 (16.6)	87 (14.6)	45 (22.3)	0.016
Previous PCI, n (%)	211 (26.5)	141 (23.7)	70 (34.7)	0.003
Previous Stroke, n (%)	84 (10.5)	59 (9.9)	25 (12.4)	0.395
Laboratory values at hospital admission				
WBC count, ×10^9^/L	7.54 ± 2.48	7.47 ± 2.59	7.74 ± 2.10	0.185
Hemoglobin, g/L	141.76 ± 15.13	141.81 ± 14.66	141.63 ± 16.49	0.884
Platelet count, ×10^9^/L	227.18 ± 60.14	225.71 ± 58.61	231.50 ± 64.39	0.238
Hs-CRP, mg/L	1.54 (0.64, 3.96)	1.53 (0.62,3.94)	1.65 (0.71, 4.10)	0.566
eGFR, mL/min	92.97 ± 16.55	92.72 ± 16.54	93.68 ± 16.62	0.479
Uric acid, umol/L	348.96 ± 98.49	351.97 ± 94.98	340.10 ± 107.93	0.139
FBG, mmol/L	6.72 ± 2.47	6.54 ± 2.24	7.24 ± 3.00	0.001
HbA1c, %	6.61 ± 1.34	6.47 ± 1.24	7.00 ± 1.55	<0.001
GA, %	16.24 ± 4.19	15.72 ± 3.76	17.79 ± 4.92	<0.001
TC (mmol/L)	3.97 ± 0.91	3.97 ± 0.90	3.94 ± 0.93	0.664
TG (mmol/L)	1.74 ± 1.22	1.77 ± 1.17	1.64 ± 1.34	0.200
LDL-C (mmol/L)	2.39 ± 0.81	2.34 ± 0.74	2.54 ± 0.97	0.002
HDL-C (mmol/L)	1.05 ± 0.25	1.05 ± 0.25	1.06 ± 0.26	0.495
LVEF (%)	61.57 ± 7.74	61.89 ± 7.38	60.62 ± 8.66	0.043
**Angiography**				
One-vessel disease, n (%)	132 (16.6)	88 (14.8)	44 (21.8)	0.028
Multivessel/LM disease, n (%)	670 (84.1)	510 (85.7)	160 (79.2)	0.038
Chronic total occlusion, n (%)	46 (5.8)	32 (5.4)	14 (6.9)	0.520
**Intervention**				
Target vessel, n (%)				
LM	21 (2.6)	17 (2.9)	4 (2.0)	0.676
LAD	334 (41.9)	239 (40.2)	95 (47.0)	0.104
LCX	146 (18.3)	116 (19.5)	30 (14.9)	0.171
RCA	291 (36.5)	219 (36.8)	72 (35.6)	0.832
Multiple stents (≥2)	290 (36.4)	198 (33.3)	92 (45.5)	0.002
Total length of stents, mm/patients	37.35 ± 23.31	35.67 ± 22.17	42.28 ± 25.82	<0.001
Minimal stent diameter, mm	2.95 ± 1.27	2.99 ± 1.45	2.82 ± 0.43	0.106
DES-sirolimus, n (%)	424 (53.2)	317 (53.3)	107 (53.0)	0.940
DES-zotarolimus, n (%)	162 (20.3)	122 (20.5)	40 (19.8)	0.910
DES-everolimus, n (%)	208 (26.1)	154 (25.9)	54 (26.7)	0.885
**Clinical diagnosis**				
STEMI, n (%)	68 (8.5)	56 (9.4)	12 (5.9)	
NSTEMI, n (%)	69 (8.7)	55 (9.2)	14 (6.9)	
UA, n (%)	644 (80.8)	474 (79.7)	170 (84.2)	
**Medications in hospital, n (%)**				
Aspirin	797 (100.0)	595 (100.0)	202 (100.0)	>0.99
Clopidogrel/Ticagrelor	797 (100.0)	595 (100.0)	202 (100.0)	>0.99
Statin	797 (100.0)	595 (100.0)	202 (100.0)	>0.99
β-block	507 (63.6)	378 (63.5)	129 (63.9)	0.932
ACEI/ARB	351 (44.0)	265 (44.5)	86 (42.6)	0.686
Insulin	71 (8.9)	47 (7.9)	24 (11.9)	0.116

BMI, body mass index; MI, myocardial infarction; PCI, percutaneous coronary intervention; WBC, white blood cell; Hs-CRP, high sensitivity c-reactive protein; eGFR, estimated glomerular filtration rate; FBG, fasting blood glucose; HbA1c, glycosylated hemoglobin A1c; GA, glycated albumin; TC, total cholesterol; TG, triglycerides; LDL-C, low-density lipoprotein cholesterol; HDL-C, high-density lipoprotein cholesterol; LVEF, left ventricular ejection fraction; LM, left main; LAD, left anterior descending artery; LCX, left circumflex artery; RCA, right coronary artery; DES, drug-eluting stents; STEMI, ST-segment elevation myocardial infarction; NSTEMI, non-ST-segment elevation myocardial infarction; UA, unstable angina; ACEI, angiotensin converting enzyme inhibitor; ARB, angiotensin receptor blocker; IQR, interquartile range. Values are presented as the mean ± SD, median (IQR) or number (%).

### Glycated albumin and the occurrence of drug-eluting stent-in-stent restenosis after successful percutaneous coronary intervention

**FIGURE 1:**
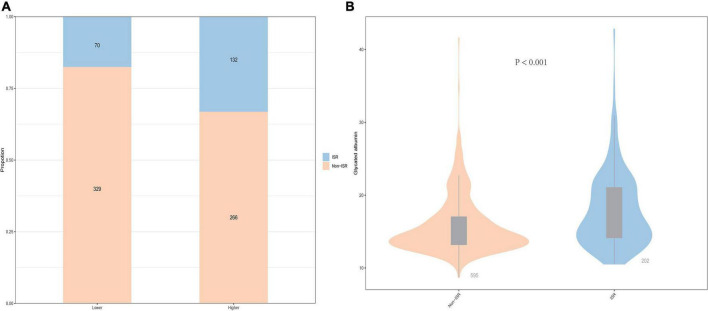
The impacts of the GA levels on the prevalence of DES-ISR **(A)** and comparison of the GA levels between the non-ISR and ISR groups **(B)** in patients with ACS. ACS, acute coronary syndrome; GA, glycated albumin; DES, drug-eluting stent; ISR, in-stent restenosis.

As shown in Figure 1A, patients with a lower GA group had 70 (8.8%) participants who were ISR and 132 (16.6%) participants were ISR in the higher GA group. Generally speaking, the prevalence of ISR is higher in the higher GA group than in the lower group. In the meantime, the violin plot of the ISR group and the non-ISR group showed the distribution of GA concentration in the two groups. As revealed in Figure 1B, the concentration of GA in the ISR group was 16.51% (14.14, 21.06), which was higher than 14.55% (13.20, 17.03) in the non-ISR group, and the difference between the two groups was statistically significant (*P* < 0.001, Figure 1B).

### Evaluate the predictive value of glycated albumin for drug-eluting stent-in-stent restenosis in univariate analysis and multivariate analysis

Univariate logistic regression analysis was performed to analyze the relationship between the GA and incidence of ISR, which is presented in Table 3. The result showed that the serum of GA, as a continuous variable, was independently associated with ISR incidence (OR = 1.12, 95% CI 1.08–1.16, *p* < 0.001). Beyond GA, age, BMI, diabetes, previous MI, previous PCI, FBG, HbA1c, LDL, LVEF, one-vessel or multivessel/LM disease, number of stents, multiple stents (≥2), total length of stents, and minimal stent diameter were risk factors for ISR in patients with ACS after PCI (all *p* < 0.05).

**TABLE 3:** Univariate logistic regression analysis for ISR.

	ISR		
	OR	95% CI	*P*-value
Age	1.02	1.00–1.04	0.04
Male	1.03	0.71–1.50	0.86
BMI	1.05	1.00–1.10	0.04
Systolic BP	1.00	0.99–1.01	0.76
Diastolic BP	0.99	0.97–1.00	0.12
Heart rate	1.00	0.98–1.02	0.90
Previous or current Smoking	1.09	0.79–1.50	0.61
Previous or current Drinking	1.01	0.72–1.43	0.95
Hypertension	0.84	0.60–1.17	0.30
Diabetes	1.9	1.37–2.63	<0.001
Dyslipidemia	1.06	0.75–1.51	0.75
Previous MI	1.67	1.12–2.50	0.01
Previous PCI	1.71	1.21–2.41	<0.001
Previous Stroke	1.28	0.78–2.11	0.33
WBC count	1.04	0.98–1.11	0.20
Hemoglobin	0.99	0.99–1.01	0.88
Platelet count	1.00	0.99–1.01	0.24
Hs-CRP	1.01	0.98–1.04	0.43
eGFR	1.00	0.99–1.01	0.48
Uric acid	1.00	0.99–1.00	0.14
FBG	1.11	1.04–1.18	<0.001
HbA1c	1.31	1.17–1.47	<0.001
GA	1.12	1.08–1.16	<0.001
TC	0.96	0.81–1.15	0.66
TG	0.91	0.78–1.05	0.20
LDL-C	1.36	1.12–1.65	<0.001
HDL-C	1.25	0.66–2.34	0.49
LVEF	0.98	0.96–1.00	0.04
One-vessel disease	1.6	1.07–2.40	0.02
Multivessel/LM disease	0.63	0.42–0.96	0.03
Chronic total occlusion	1.31	0.68–2.51	0.41
LM	0.69	0.23–2.07	0.50
LAD	1.32	0.96–1.82	0.09
LCX	0.72	0.46–1.12	0.14
RCA	0.95	0.68–1.33	0.77
Number of stents	1.44	1.16–1.80	<0.001
Multiple stents (≥2)	1.68	1.21–2.32	<0.001
Total length of stents	1.01	1.00–1.02	<0.001
Minimal stent diameter	0.63	0.44–0.89	0.01
DES-sirolimus	0.99	0.72–1.36	0.94
DES-zotarolimus	0.96	0.64–1.43	0.83
DES-everolimus	1.04	0.73–1.50	0.81
Aspirin	NA	NA–NA	NA
Clopidogrel/Ticagrelor	NA	NA–NA	NA
Statin	NA	NA–NA	NA
β-block	1.01	0.73–1.41	0.93
ACEI/ARB	0.92	0.67–1.27	0.63
Insulin	1.57	0.93–2.64	0.09

BMI, body mass index; MI, myocardial infarction; PCI, percutaneous coronary intervention; WBC, white blood cell; Hs-CRP, high sensitivity c-reactive protein; eGFR, estimated glomerular filtration rate; FBG, fasting blood glucose; HbA1c, glycosylated hemoglobin A1c; GA, glycated albumin; TC, total cholesterol; TG, triglycerides; LDL-C, low-density lipoprotein cholesterol; HDL-C, high-density lipoprotein cholesterol; LVEF, left ventricular ejection fraction; LM, left main; LAD, left anterior descending artery; LCX, left circumflex artery; RCA, right coronary artery; DES, drug-eluting stents; STEMI, ST-segment elevation myocardial infarction; NSTEMI, non-ST-segment elevation myocardial infarction; UA, unstable angina; ACEI, angiotensin converting enzyme inhibitor; ARB, angiotensin receptor blocker; OR, odds ratio; 95% CI, 95% confidence interval.

**FIGURE 2:**
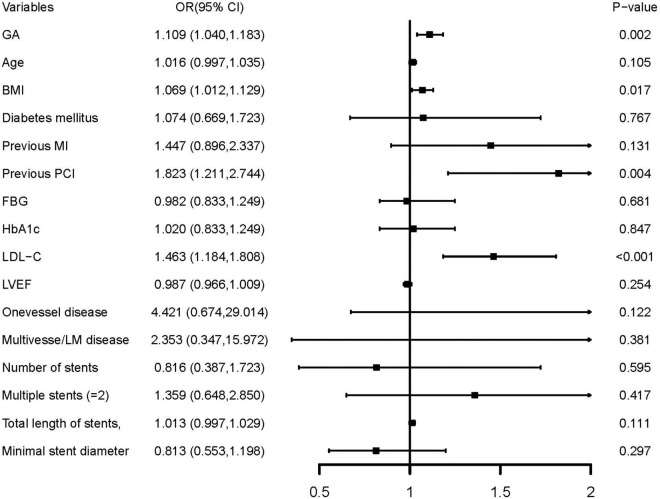
Forest plot of the multivariable logistic regression analysis model in patients with ACS evaluating the association of the GA and the risk of DES-ISR. ACS, acute coronary syndrome; GA, glycated albumin; DES, drug-eluting stent; ISR, in-stent restenosis; BMI, body mass index; MI, myocardial infarction; PCI, percutaneous coronary intervention; FBG, fasting blood glucose; HbA1c, glycosylated hemoglobin A1c; LDL-C, low-density lipoprotein cholesterol; LVEF, left ventricular ejection fraction; OR, odds ratio; CI, confidence interval.

In multivariate logistic regression models, three models, including variables that had statistical significance (*p* < 0.05), were constructed to evaluate the predictive potential of GA for the risk of DES-ISR. After adjusting for variates in the three models, regardless of the GA as a nominal or continuous variable, GA remained a significant independent risk predictor of ISR in all models (Table 4). The detailed information of Model 3 is presented in Figure 2.

**TABLE 4:** Predictive value of GA for the risk of ISR.

	As nominal variate^a^		As continuous variate^b^	
	OR (95% CI)	*P*-value	OR (95% CI)	*P*-value
Crude Model	2.332 (1.674–3.250)	<0.001	1.117 (1.076–1.160)	<0.001
Model 1	2.040 (1.349–3.085)	0.001	1.110 (1.057–1.166)	<0.001
Model 2	1.849 (1.191–2.870)	0.006	1.108 (1.040–1.180)	0.001
Model 3	1.868 (1.191–2.932)	0.007	1.109 (1.040–1.183)	0.002

Model 1: adjust for age, BMI, Diabetes. Model 2: adjusted for variates in Model 1 and Previous MI, Previous PCI, FBG, HbA1c, LDL-C, LVEF. Model 3: adjusted for variates in Model 2 and One-vessel disease, Multivessel/LM disease, Number of stents, Multiple stents (≥2), Total length of stents, Minimal stent diameter.

^a^The OR was evaluated regarding the lower median of GA as reference.

^b^The OR was evaluated by per 1-unit increase of GA. OR, odds ratio; 95% CI, 95% confidence interval.

**FIGURE 3:**
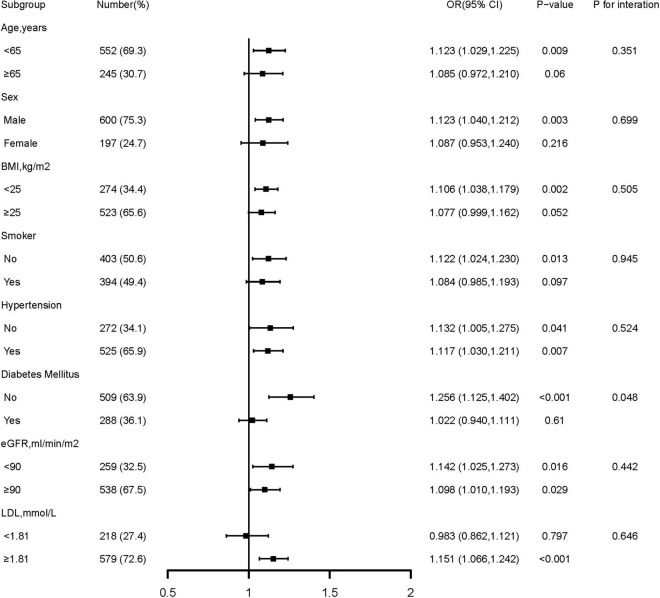
Forest plot investigating the association between GA and the prevalence of DES-ISR in different subgroups. DES, drug-eluting stent; ISR, in-stent restenosis; BMI, body mass index; eGFR, estimated glomerular filtration rate; LDL-C, low-density lipoprotein cholesterol; LVEF, left ventricular ejection fraction; OR, odds ratio; CI, confidence interval.

Further confirmation of the risk stratification value of GA for the risk of DES-ISR was performed in subgroup analysis, as presented in Figure 3. The result shows that in the subgroup of age (<65 or ≥65 years), sex (male or female), BMI (<25 or ≥25 kg/m^2^), smoking history (no or yes), hypertension (no or yes), eGFR (<90 or ≥90 ML/min/m^2^), and LDL (<1.81 or ≥1.81 mmol/L), there were no differences in the predictive power of GA for incidence of DES-ISR (all p for interaction >0.05). Interestingly, there was a slightly significant (*p* = 0.048) interaction between the GA and diabetes mellitus status concerning the risk of occurrence of DES-ISR [OR (95% CI) without diabetes mellitus 1.256 (1.125–1.402) vs. with diabetes mellitus 1.022 (0.940–1.193)].

### Incremental effects of the glycated albumin on the predictive value for drug-eluting stent-in-stent restenosis

**FIGURE 4:**
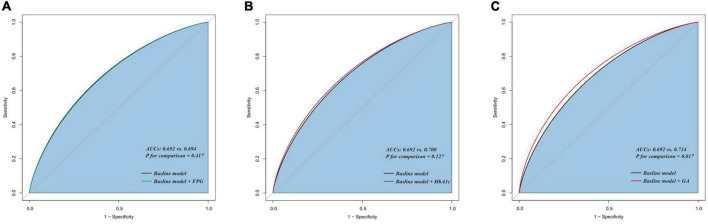
C-statistics evaluating the incremental effects of FBG, HbA1c, and GA beyond the baseline risk model. **(A)** Baseline risk model vs. +FBG; **(B)** baseline risk model vs. +HbA1c; **(C)** baseline risk model vs. +GA. AUC, the area under the curve; GA, glycated albumin; FBG, fasting blood glucose; HbA1c, glycosylated hemoglobin A1c.

The addition of GA had moderate incremental effects on the AUC obtained from the baseline risk model, which consisted of age, BMI, diabetes, previous MI, previous PCI, LDL-C, LVEF, one-vessel disease, multivessel/LM disease, number of stents, multiple stents (≥2), the total length of stents, and minimal stent diameter (AUC: baseline risk model + GA, 0.714 vs. baseline risk model, 0.692, p for comparison = 0.017) (Table 5 and Figure 4C). Moreover, adding GA to the baseline risk model could improve the reclassification and discrimination ability (category-free INR = 0.080, *P* = 0.035; IDI = 0.023, *P* < 0.001) (Table 6). However, the addition of FBG (AUC: baseline risk model + FBG, 0.694 vs. baseline risk model, 0.692, p for comparison = 0.417; category-free INR = 0.005, *P* = 0.829; IDI = 0.003, *P* = 0.229) or HbA1c (AUC: baseline risk model + HbA1c, 0.700 vs. baseline risk model, 0.692, p for comparison = 0.127; category-free INR = 0.027, *P* = 0.366; IDI = 0.009, *P* = 0.023) neither enhanced the ability of the baseline risk model to predict occurrence of ISR nor had a significant incremental effect on the reclassification and discrimination ability (Table 6 and Figures 4A,B).

**TABLE 5:** The ROC curve analysis of the GA with poor ISR.

	*AUC*	95% CI	*P*-value	*Z*-value	*P* for comparison
Baseline Model	0.692	0.651–0.733	<0.001	−	−
+FBG	0.694	0.653–0.738	<0.001	−0.8123	0.417
+HbA1c	0.700	0.659–0.743	<0.001	−1.5262	0.127
+GA	0.714	0.675–0.754	<0.001	−2.3957	0.017

BMI, body mass index; MI, myocardial infarction; PCI, percutaneous coronary intervention; LDL-C, low-density lipoprotein cholesterol; LVEF, left ventricular ejection fraction; LM, left main; FBG, fasting blood glucose; HbA1c, glycosylated hemoglobin A1c; GA, glycated albumin; AUC, area under curve; 95% CI, 95% confidence interval. The Baseline Model included age, BMI, Diabetes, Previous MI, Previous PCI, LDL-C, LVEF, One-vessel disease, Multivessel/LM disease, Number of stents, Multiple stents (≥2), Total length of stents, Minimal stent diameter.

**TABLE 6:** Category-free NRI and IDI for the incremental predictive values of various models.

	Category-free NRI			IDI		
	Index	95% CI	*P*-value	Index	95% CI	*P*-value
Baseline Model	−	−	−	−	−	−
+FBG	0.005	−0.038–0.048	0.829	0.003	−0.002–0.007	0.229
+HbA1c	0.027	−0.031–0.084	0.366	0.009	0.001–0.017	0.023
+GA	0.080	0.006–0.154	0.035	0.023	0.011–0.036	<0.001

FBG, fasting blood glucose; HbA1c, glycosylated hemoglobin A1c; GA, glycated albumin; NRI, net reclassification improvement; IDI, integrated discrimination improvement; 95% CI, 95% confidence interval.

## Discussion

This study demonstrated that subjects with higher GA levels had a significantly higher risk of developing DES-ISR than those with lower levels in patients with ACS who underwent PCI. After adjustment for confounding factors, GA, either as a continuous or nominal variable, remained an independent risk factor for DES-ISR development. Moreover, adding serum GA value to the baseline risk model could enhance the ability of the baseline risk model to predict the occurrence of DES-ISR and improve the reclassification and discrimination ability. These findings provide new perspectives on applying GA in clinical practice, particularly about early risk stratification for DES-ISR in patients with ACS.

HbA1c is widely recognized as one of the recommended diagnostic criteria for diabetes (20). It reflects the glycemic control status in 2–3 months (21). However, it does not reflect the state of blood glucose control perfectly, and it has the following limitation. First, HbA1c was influenced by the lifespan of the erythrocyte. Therefore, it does not accurately reflect blood glucose status in patients with hemoglobin variants, iron deficiency and anemia, G6-PD poverty, pregnancy, and advanced chronic kidney disease (22–24). In contrast, serum GA levels are unaffected by red blood cell lifespan, making it more accurate than HbA1c. Second, GA includes multiple glycation sites, whereas HbA1c has only one glycation site. It has been reported that the rate of glycosylation of GA is approximately 4.5 times faster than that of HbA1c (25), resulting in GA responding more rapidly than HbA1C when blood glucose changes (21). Finally, HBA1c only responds to long-term blood glucose control, while GA responds to short-term blood glucose and fluctuations in blood glucose (21, 26). Accordingly, GA was superior to HbA1c in monitoring the effect before and after drug treatment.

In recent years, many studies have shown that elevated GA levels help identify populations susceptible to cardiovascular disease. A cross-sectional study from the Japanese people has reported that serum GA levels were prominently associated with the development of carotid artery intima-media thickness, which suggests that the increased levels of serum GA can predict the progression of atherosclerosis (27, 28). Meanwhile, several studies further demonstrate that higher serum GA levels are positively associated with CVD development (29) and the severity of CAD (30, 31). Furthermore, a series of studies also confirmed that increased GA levels were associated with heart failure, impaired coronary collateralization in CTO patients (32), and adverse coronary artery remodeling (33). In addition, serum GA levels were associated with a low response to clopidogrel (34) and a collection of clinical prognoses in patients with ACS. A study from Zhang et al. (35) evaluated the prognostic value of GA in patients diagnosed with ACS who were treated with PCI and showed that elevated GA levels in the serum were associated with poor intermediate-term outcomes in patients with low-risk ACS who underwent PCI, especially in patients with preexisting diabetes. Another observational study by Liu et al. (36) followed up 2,247 patients with NSTE-ACS who were treated with PCI for 48 months and found that GA is highly correlated with cardio-cerebral events, including all-cause death, non-fatal myocardial infarction (MI), non-fatal ischemic stroke, and ischemia-induced revascularization. Extending the above findings, our results showed that GA has a positive association with DES-ISR in patients with ACS who underwent PCI. On account of our discovery, more effective management strategies to prevent the occurrence of ISR after coronary stenting are needed for these patients. For example, intraoperative endovascular imaging (IVUS or OCT) can be used to more accurately identify the need for stent implantation in coronary artery lesions. Before stent implantation, according to the imaging results, the appropriate size and length of stent can be selected. After stent implantation, IVUS or OCT can be used to check whether the stent is fully attached and adequately dilated, which can reduce the incidence of in-stent restenosis. In addition, clinicians should pay more attention to health education for such patients after surgery, and let patients strictly do secondary prevention.

Subsequently, the reliability and stability of the study results were confirmed by multivariate and subgroup analysis, both of which indicated that GA was an independent risk factor for DES-ISR. Unexpectedly, the predictive value of GA was higher in the non-diabetic subgroup than in the diabetic subgroup, and there was an interaction between the two subgroups. The exact mechanism is unclear, but it may mean that in patients without diabetes, elevated GA is closely related to the progression and occurrence of DES-ISR. This is consistent with previous studies suggesting that GA can be used as a predictor of cardiovascular events in patients without diabetes (29). Moreover, The Atherosclerosis Risk in Communities Study showed that adding serum GA levels to models with known CVD risk factors can improve the prognostic ability for CVD (14). In accordance with the previous study, our findings suggest that the prognostic of serum GA and risk of developing DES-ISR improved by the introduction of GA into the established baseline risk model, and its incremental predictive value for DES-ISR was moderate. This suggests that introducing GA into risk prediction models can better help us identify DES-ISR in clinical practice.

The potential mechanism inducing the association between elevated levels of serum GA with the development and progression of DES-ISR remains uncertain. However, it may be related to the following points: First, high serum GA was associated with a low response to clopidogrel (34), which contributes to inadequate antiplatelet, promotes platelet activation and aggregation, and increases thrombosis. Furthermore, serum GA levels reflect glycemic variability (37), which leads to endothelial dysfunction (38), and numerous studies suggest that endothelial dysfunction plays a crucial role in restenosis after coronary stent implantation (39, 40). Finally, GA induces inflammatory mediators in vessel walls and promotes the proliferation and migration of VSMCs (41), which is a significant manifestation of the pathology of ISR.

### Limitation

There are several limitations to this study. (1) This is a single-center observational study, and in addition to being unable to establish a causal relationship between GA and ISR, the power and persuasiveness of our findings are reduced. (2) Serum GA was measured only once at baseline, and changes in GA were not dynamically monitored during follow-up. Because of these constraints, serum GA levels may have been misclassified. (3) The results of this study are only for the Chinese population, and it is unknown if they apply to other racial or ethnic populations. (4) In this study, recognition of ISR was primarily based on the visual assessment by angiography rather than more accurate and informative endoluminal imaging such as IVUS or OCT. (5) This study did not exclude patients who received antidiabetic treatment before admission, which may affect the actual GA level to some extent.

## Conclusion

The GA level was significantly associated with a high risk of DES-ISR in patients with ACS who were treated with PCI. Moreover, the addition of the GA to a baseline risk model has an incremental effect on the predictive potential for DES-ISR. This conclusion needs further large-scale, randomized, multicenter studies for further confirmation.

## Data availability statement

The original contributions presented in this study are included in the article/Supplementary material, further inquiries can be directed to the corresponding author.

## Ethics statement

This study was conducted according to the Helsinki Declaration of Human Rights and approved by the Clinical Research Ethics Committee of Beijing Anzhen Hospital, Capital Medical University. Written informed consent was otained from all patients.

## Author contributions

XLL took responsibility for all aspects of the reliability and freedom from bias of the data presented and their discussed interpretation. QF and XLL took responsibility for research design. QYL and DHZ took responsibility for data collection. JHL took responsibility for the data review. All authors read and approved the final manuscript.

## Supplementary material

Supplementary Figure 1 Flowchart of the selection of study enrollment.
