# Supplementary material for: Serum glycated albumin is associated with in-stent restenosis in patients with acute coronary syndrome after percutaneous coronary intervention with drug-eluting stents: An observational study
Source: Front Cardiovasc Med. 2022 Sep 27;9:943185. doi: 10.3389/fcvm.2022.943185 (PMC9551162; doi:10.3389/fcvm.2022.943185)
Supplement: Supplementary file 1 [file Data_Sheet_1.docx]

**Table S1:** **Baseline Procedural characteristics of study population stratified by median of GA**

|  | Lower  GA(≤14.94; n = 399) | Higher  GA(>14.94; n = 398) |
| --- | --- | --- |
| Number of target vessels |  |  |
| Onevessel disease, n (%) | 64 (16.0) | 68 (17.1) |
| Multivesse/LM disease, n (%) | 339 (85.0) | 331 (83.2) |
| Chronic total occlusion, n (%) | 25 (6.3) | 21 (5.3) |
| Target lession |  |  |
| Left Main trunk, n (%) | 15 (3.8) | 6 (1.5) |
| Left anterior descending, n (%) | 162 (40.6) | 172 (43.2) |
| Left circumflex, n (%) | 66 (16.5) | 80 (20.1) |
| Right coronary artery, n (%) | 155 (38.8) | 136 (34.2) |
| Lesion Morphology |  |  |
| Type A, n (%) | 53 (13.3) | 53 (13.3) |
| Type B, n (%) | 121 (30.3) | 112 (28.1) |
| Type C, n (%) | 225 (56.4) | 233 (58.5) |
| Classification of ISR |  |  |
| Type I, n (%) | 42 (10.5) | 65 (16.3) |
| Type II, n (%) | 16 (4.0) | 33 (8.3) |
| Type III, n (%) | 5 (1.3) | 15 (3.8) |
| Type IV, n (%) | 7 (1.8) | 19 (4.8) |
| Lesion length, mm | 29.87±23.70 | 29.34±22.38 |
| Reference diameter, mm | 2.76±1.52 | 2.60±0.58 |
| Multiple stents (≥2), n (%) | 146 (36.6) | 144 (36.2) |
| Total length of stents, mm | 37.65±23.93 | 37.04±22.70 |
| Minimal stent diameter, mm | 3.04±1.68) | 2.86±0.64 |
| DES-sirolimus, n (%) | 210 (52.6) | 214 (53.8) |
| DES-zotarolimus, n (%) | 81 (20.3) | 81 (20.4) |
| DES-everolimus, n (%) | 105 (26.3) | 103 (25.9) |

**Table S2.** **The follow-up lab measures of patients with and without ISR**

|  | Total population  (n = 797) | Non-ISR group  (n = 595) | ISR group  (n = 202) | P-value |
| --- | --- | --- | --- | --- |
| WBC count, ×10^9^/L | 7.10±1.81 | 7.01±1.78 | 7.36±1.86 | 0.017 |
| Hemoglobin, g/L | 138.08±16.67 | 138.07±16.84 | 138.11±16.21 | 0.977 |
| Platelet count, ×10^9^/L | 216.95±58.32 | 214.60±56.09 | 223.88±64.08 | 0.051 |
| eGFR, mL/min | 90.99±17.60 | 91.63±17.08 | 89.10±18.98 | 0.077 |
| Uric acid, umol/L | 350.35±97.83 | 349.97±96.59 | 351.44±101.63 | 0.854 |
| FBG, mmol/L | 6.56±2.34 | 6.45±2.25 | 6.87±2.58 | 0.026 |
| HbA1c, % | 6.57±1.28 | 6.48±1.17 | 6.82±1.55 | 0.001 |
| GA, % | 15.08±3.74 | 14.80±3.61 | 15.91±4.01 | <0.001 |
| TC (mmol/L) | 3.52±0.84 | 3.55±0.85 | 3.44±0.82 | 0.133 |
| TG (mmol/L) | 1.85±1.00 | 1.85±1.00 | 1.86±1.01 | 0.833 |
| LDL-C (mmol/L) | 1.86±0.70 | 1.86±0.71 | 1.88±0.68 | 0.781 |
| HDL-C (mmol/L) | 0.99±0.22 | 0.99±0.22 | 0.98±0.21 | 0.521 |

Abbreviations: BMI, body mass index; MI, myocardial infarction; PCI, percutaneous coronary intervention; WBC, white blood cell; eGFR, estimated glomerular filtration rate; FBG, fasting blood glucose; HbA1c, glycosylated hemoglobin A1c; GA, glycated albumin; TC, total cholesterol; TG, triglycerides; LDL-C, low-density lipoprotein cholesterol; HDL-C, high-density lipoprotein cholesterol.

Note: Values are presented as the mean±SD, median (IQR) or number (%).

**Table S3: Baseline Procedural characteristics of study population with and without ISR**

|  | Non-ISR group  (n = 595) | ISR group  (n = 202) |
| --- | --- | --- |
| Number of target vessels | 88 (14.8) | 44 (21.8) |
| Onevessel disease, n (%) | 510 (85.7) | 160 (79.2) |
| Multivesse/LM disease, n (%) | 32 (5.4) | 14 (6.9) |
| Chronic total occlusion, n (%) | 88 (14.8) | 44 (21.8) |
| Target lession |  |  |
| Left Main trunk, n (%) | 17 (2.9) | 4 (2.0) |
| Left anterior descending, n (%) | 239 (40.2) | 95 (47.0) |
| Left circumflex, n (%) | 116 (19.5) | 30 (14.9) |
| Right coronary artery, n (%) | 219 (36.8) | 72 (35.6) |
| Lesion Morphology |  |  |
| Type A, n (%) | 93 (15.6) | 13 (6.4) |
| Type B, n (%) | 175 (29.4) | 58 (28.7) |
| Type C, n (%) | 327 (55.0) | 131 (64.9) |
| Classification of ISR |  |  |
| Type I, n (%) | 0 (0.0) | 107 (53.0) |
| Type II, n (%) | 0 (0.0) | 49 (24.3) |
| Type III, n (%) | 0 (0.0) | 20 (9.9) |
| Type IV, n (%) | 0 (0.0) | 26 (12.9) |
| Lesion length, mm | 27.97±21.85 | 34.41±25.68 |
| Reference diameter, mm | 2.72±1.32 | 2.57±0.39 |
| Multiple stents (≥2), n (%) | 198 (33.3) | 92 (45.5) |
| Total length of stents, mm | 35.67±22.17 | 42.28±25.82 |
| Minimal stent diameter, mm | 2.99±1.45 | 2.82±0.43 |
| DES-sirolimus, n (%) | 317 (53.3) | 107 (53.0) |
| DES-zotarolimus, n (%) | 122 (20.5) | 40 (19.8) |
| DES-everolimus, n (%) | 154 (25.9) | 54 (26.7) |
