# Supplementary figures and images for: Serum glycated albumin is associated with in-stent restenosis in patients with acute coronary syndrome after percutaneous coronary intervention with drug-eluting stents: An observational study
Source: Front Cardiovasc Med. 2022 Sep 27;9:943185. doi: 10.3389/fcvm.2022.943185 (PMC9551162; doi:10.3389/fcvm.2022.943185)

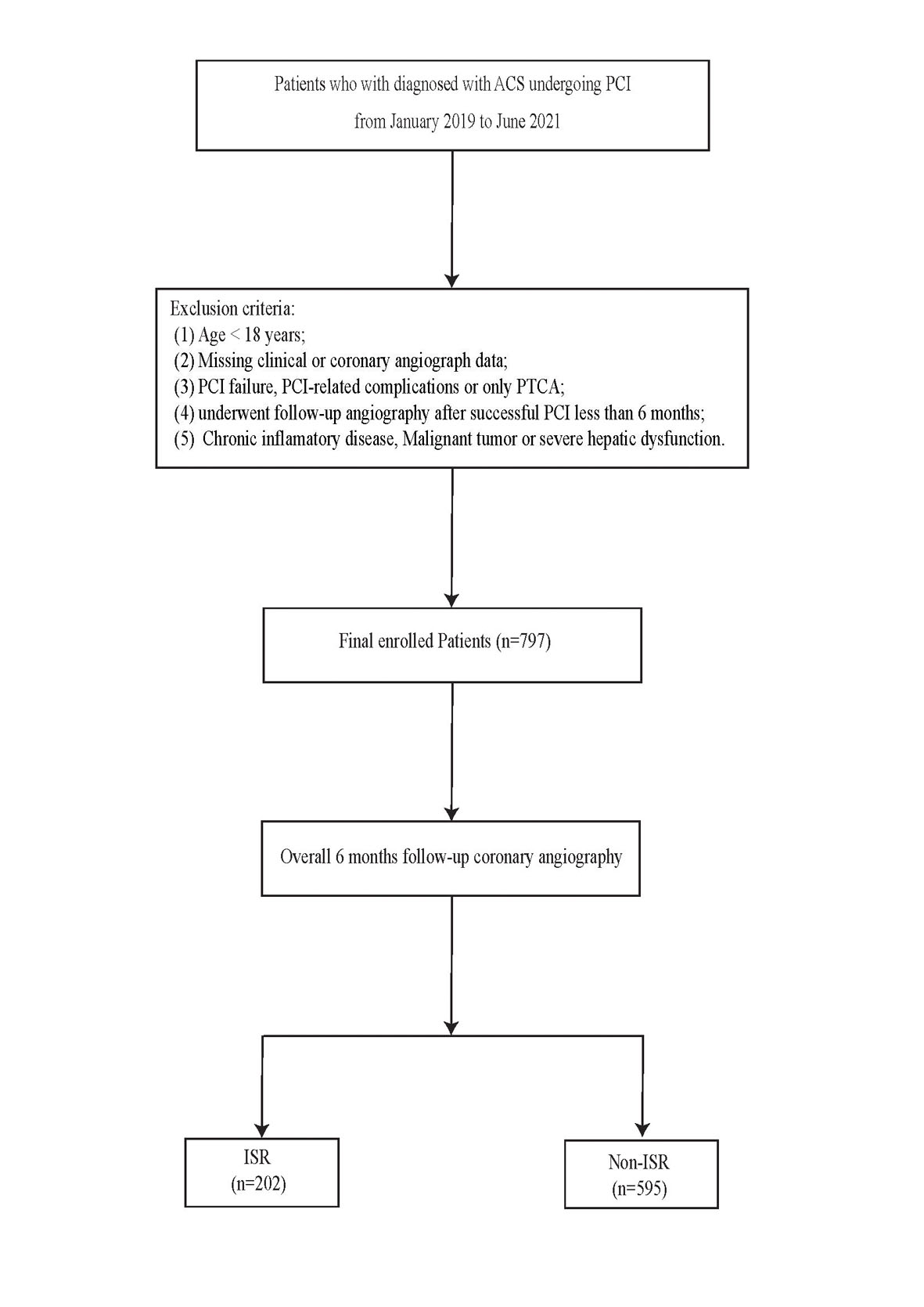

Supplement: Supplementary Figure 1 — Flowchart of the selection of study enrollment. [file Image_1.jpeg]
